# QTL-seq analysis identified the genomic regions of plant height and days to heading in high-latitude rice

**DOI:** 10.3389/fgene.2024.1305681

**Published:** 2024-02-14

**Authors:** Rongsheng Wang, Kun Li, Wei Zhang, Hui Liu, Yongqing Tao, Yuming Liu, Guohua Ding, Guang Yang, Yuanhang Zhou, Jiayou Wang, Licheng Wu, Baohai Liu, Fengchen Mu

**Affiliations:** ^1^ Biotechnology Research Institute, Heilongjiang Academy of Agricultural Sciences, Harbin, Heilongjiang, China; ^2^ Heilongjiang Laboratory of Crop and Livestock Molecular Breeding, Harbin, Heilongjiang, China; ^3^ Heilongjiang Engineering and Technology Research Center of Rice Molecular Breeding, Harbin, Heilongjiang, China; ^4^ Heilongjiang Academy of Agricultural Sciences, Harbin, Heilongjiang, China

**Keywords:** rice, days to heading, plant height, QTL-seq, bulked segregant analysis

## Abstract

**Introduction:** Rice (*Oryza sativa* L.) is one of the most extensive crops in the world. China’s Heilongjiang Province is the northernmost rice-growing region in the world. However, rice cultivars suitable for growth in low-latitude regions may not mature normally due to their distinct climate and short frost-free period. It is necessary to precisely determine the frost-free period for each region to make the best use of the rice growth stage so as to ensure the maturity and yield of different rice cultivars in Heilongjiang Province. The time span of the heading stage is a key parameter for evaluating the adaptability of a rice cultivar to a specific rice-growing region. Given the above facts, it is of high importance to study the associated genes and sites controlling days to heading (DH) and plant height (PH) of rice in Heilongjiang Province. Bulked segregant analysis (BSA) combined with high-throughput sequencing can effectively exclude interferences from background genomic differences, making it suitable for analyzing the associated sites of complex agronomic traits in early generations.

**Methods:** In this study, an F_3_ segregating population was obtained by crossing two main cultivars that are grown under different temperatures and day-light conditions in Heilongjiang. Two pools of extreme phenotypes were built for the DH and PH of the population. For SNP and InDel variants obtained from whole-genome resequencing in the pools, an association analysis was performed using the Euclidean distance (ED) algorithm and the SNP/InDel index algorithm.

**Results:** The intersection of SNP and InDel regions associated with the phenotypes was considered to obtain the final associated sites. After excluding interferences from the cloned genes on chromosomes 2 and 7, a total length of 6.34 Mb on chromosomes 1, 3, and 10 and 3.16 Mb on chromosomes 1 and 10 were left associated with PH and DH, respectively. Then, we performed a gene annotation analysis for candidate genes in the remaining regions using multiple genome annotation databases. Our research provides basic data for subsequent gene mapping and cloning.

**Discussion:** By mining more genetic loci associated with the days to heading and plant height of rice, we may provide abundant genetic resources for refined molecular breeding in Heilongjiang Province.

## Introduction

Rice (*Oryza sativa* L.) is one of the most important food crops in the world. To satisfy the varying dietary needs and consumer demands of larger populations, people have been continuously imposing artificial selection on rice and acclimatizing the cultivar. Rice originates in low-latitude regions with short daylight durations. At present, some rice cultivars can adapt to unique light and temperature conditions in high-latitude regions (Xing and Zhang, 2010; Wing, Purugganan, and Zhang, 2018). China’s Heilongjiang Province is the world’s most important high-latitude rice-planting region, with an annual sowing area of over 3.8 million ha, which accounts for more than 12% of China’s total rice-planting area (Lu et al., 2017). The long sunshine duration and unique characteristics of accumulated temperature during the rice growth stage in high-latitude regions constitute directional selection pressures on regulatory genes controlling the main cultivars' growth stage and yield. As a result, the genes and regulatory pathways for a photoperiodic response that control the heading and seed-setting of rice are unique to local light and temperature conditions (Matsubara et al., 2014).

At present, a variety of genes related to rice heading date have been cloned. For example, the *Ghd7* (Xue et al., 2008; Liu et al., 2015) and *Hd1* genes (Yano, 2000; Griffiths et al., 2003) respond to long- and short-sunshine durations and regulate premature and postponed heading, respectively. The *DTH7* (Gao et al., 2014), *Hd3a* (Kojima et al., 2002), and *DTH8* genes (Wei et al., 2010) are also identified as key regulatory genes of the photoperiod response in rice. Cloning of the above genes is a crucial part of the investigation into the regulatory mechanisms of photoperiod and yield. The cloned genes constitute important genetic resources for trait improvement in rice during the growth stage (Shrestha et al., 2014). In addition, many quantitative trait loci (QTL) related to the rice growth stage have been localized. However, the phenotype-associated genes and regulatory mechanisms remain to be further understood (Matsubara et al., 2008; J; Li et al., 2018). Studies conducted on regulatory genes for the rice growth stage have shown that as heading comes earlier or later than normal under the regulatory action of relevant genes, the plant height and yield of rice either decrease or increase (Yan et al., 2013; Q; Wang et al., 2021). The above facts indicate the pleiotropy of the regulatory genes that are involved in various regulatory pathways governing rice growth and development. Days to heading and yield-related traits are complex quantitative (polygenic) traits. More minor loci need to be mined, and the genetic regulation mechanism may be understood more thoroughly using advanced analytical tools and methodologies in genetic populations constructed with various techniques. These efforts are important for providing abundant candidate loci for the molecular breeding of rice.

Rapid developments in molecular marker technology have facilitated gene mapping studies. It may be combined with several other techniques to result in even more methods for gene mapping. Bulked segregant analysis (BSA) was first proposed for the localization and screening of the downy mildew resistance gene in the F_2_ segregating population of lettuce. BSA involves constructing two pools of individuals with extreme phenotypes and transforming a pair of phenotypic traits in parents into variations in a single-DNA region in the pool of individuals. Next, DNA markers in the pool are screened based on their differences in frequency so as to obtain linked markers for target traits (Silva et al., 2020; Michelmore et al., 1991). There is no need to create a near-isogenic line to quickly exclude interferences caused by a genetic background from the target traits. However, during its early applications, BSA was only adequate for mapping genes associated with dominant or extreme phenotypes due to the limitations of marker density and population size. BSA is less suitable for mining complex QTLs, especially minor effect traits. Progress in high-throughput sequencing and cost reduction has made the combination of high-throughput sequencing and BSA a novel strategy for gene mapping. High-density SNP and InDel markers allow for the rapid and precise localization of target genes (Abe et al., 2012). So far, this strategy has been widely applied to precise the localization of QTLs, and several analytical algorithms for high-throughput sequencing data have been proposed.

The SNP-index algorithm proposed in earlier years uses significant differences in the genotypic frequencies of rice in the pool for the association analysis of markers (Takagi et al., 2013). Typically, Δ (SNP-index) is calculated as an analytical parameter. The greater the association between the genetic locus and the phenotype, the greater the differences in genotypic frequency at this locus, that is, the closer the value is to 1. During the analysis, the Δ (SNP-index) value of the extreme phenotype pool and the Δ (SNP-index) value on the same chromosome are fitted by regression. The resulting association threshold is used to identify the associated regions. The Euclidean distance (ED) method is another approach for similar analytical purposes. The ED method calculates the differences in mutation frequencies at each locus in the pool and ranks the results in ascending order using the quantile method. ED values are fitted, and the value higher than 99% of those of SNP markers is chosen as the threshold for screening. Finally, we can obtain the linkage relationship between the markers and target regions (Abe et al., 2012; Fekih et al., 2013). The above two algorithms are different in that the ED algorithm can effectively remove background noises without the need for resequencing data from parents. Moreover, the ED algorithm is less costly and more efficient. An appropriate method can be chosen between the two, or the two methods may be used in combination based on how the population is constructed and the population size so as to increase the detection rate of associated loci (Hill et al., 2013).

This study was intended to mine genetic loci associated with the days to heading and plant height of rice cultivars in high-latitude regions. The results can further contribute research-based knowledge to the study of precise molecular breeding and gene functional analysis. For the above purpose, we crossed two japonica rice cultivars with distinct phenotypic differences, both grown in Heilongjiang Province. Using the F_2_ segregating population, we further constructed the F_3_ segregating population, consisting of 299 individuals. Data on plant height and days to heading were collected in the F_3_ segregating population, and pools of extreme phenotypes were constructed for each. Resequencing data for each pool of extreme phenotypes was analyzed using the ED and SNP-index algorithms. We finally identified QTLs associated with the days to heading and plant height of rice in high-latitude regions. The results provide important clues for analyzing the regulatory mechanisms of rice growth and development and for guiding molecular breeding practices.

## Materials and methods

### Population development and field management

A segregating population was constructed using two main rice cultivars grown in Heilongjiang, which differed in the growth stage. The growth stage of Hua Chuan-Xiang (HC) lasts for approximately 135 days, and the main stem has 12 leaves. Songgeng 22 (SG) is the main late-maturing cultivar in Heilongjiang Province. Its growth stage lasts for approximately 144 days, and the main stem has 14 leaves. The two cultivars were crossed, with HC as the female parent and SG as the male parent. The grains of the F_2_ population were mixed-harvest and were randomly picked for germination and seedling growth. The rice seedling was transplanted with one plant per hole in the test field in Minzhu Town under the Heilongjiang Academy of Agricultural Sciences during the growing season in 2021. The row spacing was 30 cm, and the plant spacing was 12 cm. A phenotypic analysis and experimental study were conducted for the F_3_ segregating population. Other measures of paddy field management were consistent with local standards.

### Sample collection and phenotypic analysis

Days to heading and plant height were surveyed for each individual in the population. Days to heading (d) was defined as the number of days from the soaking of rice grains to the emergence of the main spike at the top of leaf sheaths. Plant height (cm) was the distance from the ground to the spike top of a single plant after maturation. The results of a phenotypic survey were arranged in descending order. From all individuals in the population, 10% were selected to create pools of high- and low-phenotypic values. Equal amounts of leaves were evenly collected from individuals in each pool using a punch. The leaf samples were mixed together as samples for the corresponding pool. Meanwhile, leaves were collected from two parents. DNA extraction was performed from leaves using the CTAB method (Masoodi et al., 2021). The concentration of the extracted DNA was detected using the Qubit fluorometer (Thermo Fisher Scientific). The subsequent resequencing procedures were performed after ensuring that the DNA samples met the quality requirements for library construction. Statistical analysis and plotting of phenotypic data were conducted using the R programming language (Milano, 2019).

### Resequencing and data analysis

Resequencing was carried out in accordance with the standard protocol established by Illumina. After passing the detections, the DNA samples were used for library construction. Those passing the quality control were further sequenced on the Illumina HiSeq platform. The raw reads were subjected to base identification using BCL to FASTQ 1.8.4 (https://support.illumina.com.cn/downloads/bcl2fastq_conversion_software_184.html) to obtain paired-end sequencing data, with the read length being 150 bp. Adapter and low-quality sequences (base pairs with a quality score ≦10, accounting for over 50% of the entire read) were first trimmed. If N bases accounted for over 10% of the entire read, the read pair was removed. The filtered reads were aligned to the reference genome of Nipponbare [MSU Rice Genome Annotation Project (Release 7) (Kawahara et al., 2013)] using BWA software (Li and Durbin, 2009). Duplicate reads were excluded from aligned reads using SAMtools (v1.9) (Li, 2011).

The filtered reads were grouped by the phenotype under study. Each group consisted of four samples (one sample from each parent and one from the dominant and recessive phenotypic pools). The four samples in each of the two phenotypic groups were subjected to variation detection and filtering. SNP and small InDel (1–50 bp) variants were detected using the GATK package (McKenna et al., 2010). The identified SNPs near InDel within 5 bp and the adjacent InDels within 10 bp were filtered using the script vcfutils.pl provided by BCFtools. The number of variants within 5 bp should not exceed 2. In addition, those with a quality score <30 and a quality/depth <2.0 were removed (H. Li, 2011). Other filtering parameters were default values specified in the GATK Best Practices to obtain high-quality SNPs and InDels for association analysis (Reumers et al., 2012).

### Association analysis based on the SNP index and ED algorithms

SNP and InDel variants were filtered before the association analysis to remove SNPs with several genotypes; SNPs with support depth below 4 and SNPs on recessive alleles that were not inherited from recessive parents were removed. The Euclidean distance was calculated as follows: D = $\sqrt{\sum {\left(N_{\text{mut}}-N_{\text{wt}}\right)}^{2}}$ , which is the sum of the squares of the difference between the frequency of each base in the mutant pool and that in the wild-type pool. The coverage depths were determined for SNPs with genotypic differences between the pools. ED values were calculated at each locus, and those of non-target loci approached 0 in theory. The ED value to the fifth power was considered the association value to eliminate background noises. ED values were fitted using the distance method (Hill et al., 2013). The median + 3 SD of the fitted value at all loci was chosen as the threshold for association analysis.

The SNP index is a method used to identify significant differences in genotypic frequency between the pools (Takagi et al., 2013), and ΔSNP-index = M_aa_/(M_aa_ + P_aa_)–M_ab_/(M_ab_ + P_ab_), where M_aa_ and M_ab_ represent the depths of reads obtained from the female parent in the pools aa and ab, respectively, and P_aa_ and P_ab_ represent the depths of reads obtained from the male parent in the pools aa and ab, respectively. It can be known from the above formula that the stronger the association between SNP markers and the trait, the closer the ΔSNP-index value is to 1. The method and threshold setting for small InDels were the same as those for SNPs.

### Annotation of mutant loci and candidate genes

Mutant sites were annotated using SnpEff software. The mutant sites were localized based on the positions of SNPs and small InDels identified in the whole genome or in genotype-associated regions mapped to the reference genome. The impacts of these mutant sites were also predicted (Cingolani et al., 2012). For all genes within the candidate regions, deep annotation was performed using the Gene Ontology (GO) and Kyoto Encyclopedia of Genes and Genomes (KEGG) databases. First, gene ID conversion was performed using plant gene set enrichment analysis (GSEA) (http://structuralbiology.cau.edu.cn/PlantGSEA/analysis.php), that is, MSU IDs were converted into UniProt IDs, followed by GO and KEGG enrichment analyses using database for annotation, visualization, and integrated discovery (DAVID; https://david.ncifcrf.gov/) (Ashburner et al., 2000; Kanehisa et al., 2004). The species was annotated as the *O. sativa* japonica group. A visualization diagram was plotted using R/ggplot2 based on the results (Wickham, 2016).

### Polymorphism analysis of known genes in the associated regions

Based on references and database searches, localized and cloned genes highly linked to days to heading and plant height were retrieved for the phenotype-associated regions. Primers were designed based on sequences near each gene-coding region or SNP locus. The amplified products were sequenced. The sequences were assembled and aligned using DNAMAN 6.0.3 (Lynnon Corporation, Quebec, Canada). The influence of known genes on candidate regions for association analysis was excluded.

## Results

### Phenotypic variations in the population and the construction of extreme phenotypic pools

**FIGURE 1:**
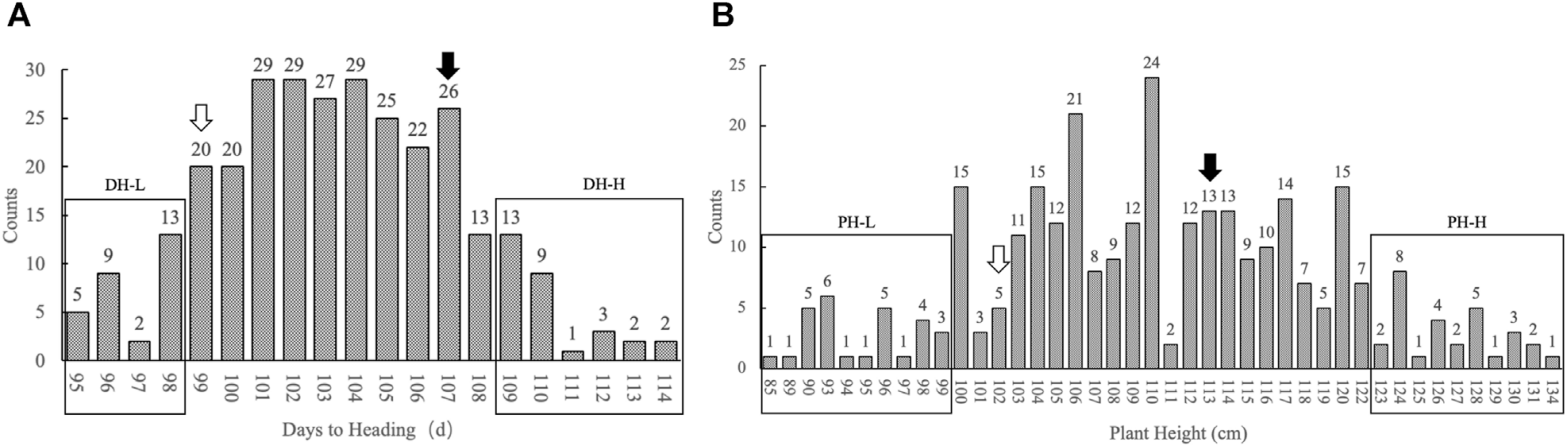
Histogram of the number of individuals with different phenotypes in the population. **(A)** The *x*-axis represents days to heading from low to high; the *y*-axis represents the number of individuals with different days to heading, marked by figures on top of each column. **(B)** The *x*-axis represents plant height from low to high; the *y*-axis represents the number of individuals with different plant heights, marked by figures on top of each column. The rectangular box represents the distribution of individuals selected from each pool. The hollow arrow represents the position of the phenotype of the parent HC in the population. The black solid arrow represents the position of the phenotype of the parent SG in the population.

The F_3_ segregating population comprised 299 plants. The days to heading were continuously distributed in the population, the shortest being 95 d and the longest being 114 d, with an average of 105 d. Statistical analysis showed that the number of individuals basically followed a normal distribution in terms of days to heading. The numbers of individuals with two extreme heading phenotypes (either too early or too late) were close. Therefore, the F_3_ population was suitable for constructing the pool of extreme heading phenotypes. Individuals whose heading stage was obtained in the first 4 days constituted the days to heading lower (DH-L) pool. This pool comprised 29 individuals, whose days to heading ranged from 95 to 98 d. Individuals whose heading stage was obtained in the last 6 days constituted the days to heading higher (DH-H) pool. This pool comprised 30 individuals, whose days to heading ranged from 109 to 114 d. The plant heights of the population basically followed a normal distribution, with the shortest individual being 85 cm and the tallest being 134 cm, with an average of 110 cm. Twenty-eight shortest individuals constituted the plant height lower (PH-L) pool, with the plant height ranging from 85– to 99 cm. Twenty-nine highest individuals constituted the plant height higher (PH-H) pool, with the plant height ranging from 123 to 134 cm. The individuals in each pool accounted for approximately 10% of the total individuals (Figure 1).

### Whole-genome resequencing of the parent, pool, and variant analysis

Six samples, namely, one from each of the maternal parent, paternal parent, DH-L pool, DH-H pool, PH-L pool, and PH-H pool, were subjected to whole-genome resequencing, which yielded 118.63 Gbp of clean reads, with the average Q30 being 95.74% and the average GC-content of the sample being 39.71%. An average of 96.52% of paired-end reads were mapped to the reference genome, where the average coverage depth of the sample was approximately 44X; bases covered at a depth of 10X and above accounted for 95.36% of all bases in the reference genome (Table 1). The reads from the four samples in the plant height phenotypic group were aligned to the reference genome, and the average number of SNPs in the pool was 638,660. The transition-to-transversion ratio was approximately 2.54, and the heterozygosity ratio for SNPs in the pool was 87.94%. The heterozygosity ratio for InDel in the CDS region was 84.19% on an average basis. The total average number of InDels in the genome was 143,283. The distributions and percentages of SNPs and InDels in the four samples from the days to heading phenotypic group were similar to those from the plant height phenotypic group (Supplementary Table S1, Supplementary Figure S1). The above results indicated the high purity of the samples and the differences compared with the reference genome. The heterozygosity ratio was higher in the pools than in the parents. The transition-to-transversion ratio agreed with the species law, indicating that the parental selection of the species and pool construction conformed to the analytical requirements. The parents and constructed pools were highly representative of the population.

**TABLE 1:** Statistics of sequencing data for the parents and pools.

ID	Clean reads	Clean base	Q30 (%)	GC (%)	Mapped (%)	Properly (%)	Average depth	1X coverage ratio (%)	10X coverage ratio (%)
HC	48,445,198	14,263,201,994	96.23	38.21	99.50	93.09	31	97.63	88.40
SG	50,128,662	14,847,459,750	95.73	39.16	99.34	95.71	31	97.44	92.85
PH-L	74,876,915	22,189,665,286	95.92	41.08	99.36	97.80	51	99.52	98.30
PH-H	74,044,813	21,889,188,260	95.25	39.22	99.29	97.35	48	99.05	96.73
DH-L	77,911,371	23,108,044,930	95.44	40.39	99.24	97.64	52	99.44	98.21
DH-H	75,536,000	22,336,636,730	95.85	40.21	99.34	97.51	51	99.17	97.67

**Note:** ID, sample name; clean reads, number of read pairs after filtering, with a paired-end read defined as 1 read; clean base, number of bases after filtering; Q30, percentage of bases with a quality score of 30 or higher; GC, percentage of G and C bases on a DNA molecule; mapped, percentage of clean reads mapped to the reference genome to total clean reads; properly mapped, paired-end reads are all localized to the reference genome and the distance matches the length distribution of the sequenced fragment; average depth, average coverage depth of the sample; %X coverage ratio, percentage of bases covered at the specified coverage depth and above to total bases in the reference genome.

SNPs and InDels were analyzed for the four samples (one from each of paternal, maternal, phenotypic lower pool, and phenotype higher pool) for each phenotype. There were 146,153 SNPs and 38,222 InDels between the PH pools. There were 126,347 SNPs and 33,304 InDels between the DH pools. All of the differential loci were subjected to gene annotation. The position of each locus in the genome was analyzed statistically based on its type (Supplementary Tables S2, S3). These differential loci were further filtered (Reumers et al., 2012) to obtain high-quality loci for association analysis, resulting in 382,591 SNPs and 79,491 InDels associated with plant height and 386,439 SNP and 80,591 InDels associated with days to heading.

### QTL-seq-based association analysis and candidate locus annotation

**FIGURE 2:**
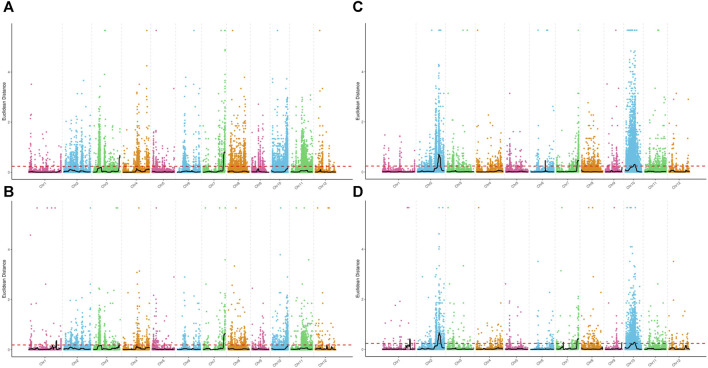
Distribution of ED values at each locus on the chromosomes. **(A)** ED values of each SNP associated with the plant height phenotype, **(B)** ED values of each small InDel associated with the plant height phenotype, **(C)** ED values of each SNP associated with the days to heading phenotype, and **(D)** ED values of each small InDel associated with the days to heading phenotype. The *x*-axis represents the name of each chromosome; the colored dots represent the ED values of each locus; the black line is the fitted ED value; and the red dotted line represents the threshold for significant association.

Association analysis was performed for SNP and InDel sites in the plant height phenotypic pool. The association threshold for the ED algorithm was 0.24 and 0.19 for SNP and InDel analysis, respectively (Figures 2A, B). The confidence level was set to 0.99 for the SNP-index algorithm (Supplementary Figures S2A, B). The phenotype-associated regions identified by the SNP-index algorithm are shown in Supplementary Table S4. The intersection of SNP and InDel regions associated with phenotypes as identified by the two algorithms was considered. Finally, we obtained 5 associated regions on 4 chromosomes with a total length of 8.72 Mb, containing 1,323 genes. The polymorphic sites were searched in the above regions, resulting in 11,623 SNPs and 2,441 InDels (Table 2). These polymorphic sites were annotated. There were 729 nonsynonymous mutations, 42 frameshift mutations in parents, and 39 nonsynonymous mutations in the pool (Supplementary Tables S5, S6). These mutations were very likely to be linked to the plant height phenotype, and the related genes deserve further annotation and analysis.

**TABLE 2:** Details of the final associated regions and statistical summary of the numbers of polymorphic sites and genes in the associated regions.

Traits	Chromosome	Start	End	Size (Mb)	Gene number	SNP	InDel	Genes contained
PH	Chr1	36,420,000	37,240,000	0.82	113	3	—	—
PH	Chr3	6,060,000	11,030,000	4.97	757	9,514	1,926	—
PH	Chr3	34,420,000	34,490,000	0.07	11	—	—	—
PH	Chr7	27,310,000	29,690,000	2.38	373	256	108	*OsCOL13* (Sheng et al., 2016) and *DTH7* (Gao et al., 2014)
PH	Chr10	21,460,000	21,940,000	0.48	69	1,850	407	—
DH	Chr1	36,420,000	37,240,000	0.82	113	3	1	—
DH	Chr2	27,240,000	31,070,000	3.83	584	888	287	*SID1* (Deng et al., 2017), *DTH2* (Wu et al., 2013), and *Ghd2* (Liu et al., 2016)
DH	Chr7	27,520,000	29,690,000	2.17	338	256	105	*OsCOL13* (Sheng et al., 2016) and *DTH7* (Gao et al., 2014)
DH	Chr10	11,130,000	13,470,000	2.34	349	10,106	2,006	—

For the days to heading phenotype, the association threshold was set to 0.24 for SNP and InDel analysis using the ED algorithm (Figures 2C, D). The confidence level was also set to 0.99 for the SNP-index algorithm (Supplementary Figures S2C, D). There were 7 associated regions identified at SNP sites and 10 associated regions at InDel sites (Supplementary Table S4). An intersection of associated regions identified by the two methods was considered. Finally, we obtained 4 shared associated regions on 4 chromosomes with a total length of 9.16 Mb, containing 1,384 genes. The polymorphic analysis of these regions identified 11,253 SNPs and 2,399 InDels (Table 2). The annotation analysis showed that among the polymorphic sites in parents, there were 918 nonsynonymous mutations and 78 frameshift mutations. In the pools, 137 nonsynonymous mutations and 10 frameshift mutations were identified. The above loci deserve further investigation, including gene annotation and expression analysis (Supplementary Tables S5, S6).

An intersection of the final regions associated with plant height and days to heading phenotype was considered, resulting in two shared associated regions totaling 2.99 Mb in length on chromosomes 1 and 7. These associated regions contained 451 genes. It is very likely that the pleiotropic loci related to plant height and days to heading were localized to these two regions.

### Polymorphism analysis of known genes in the associated regions

**FIGURE 3:**
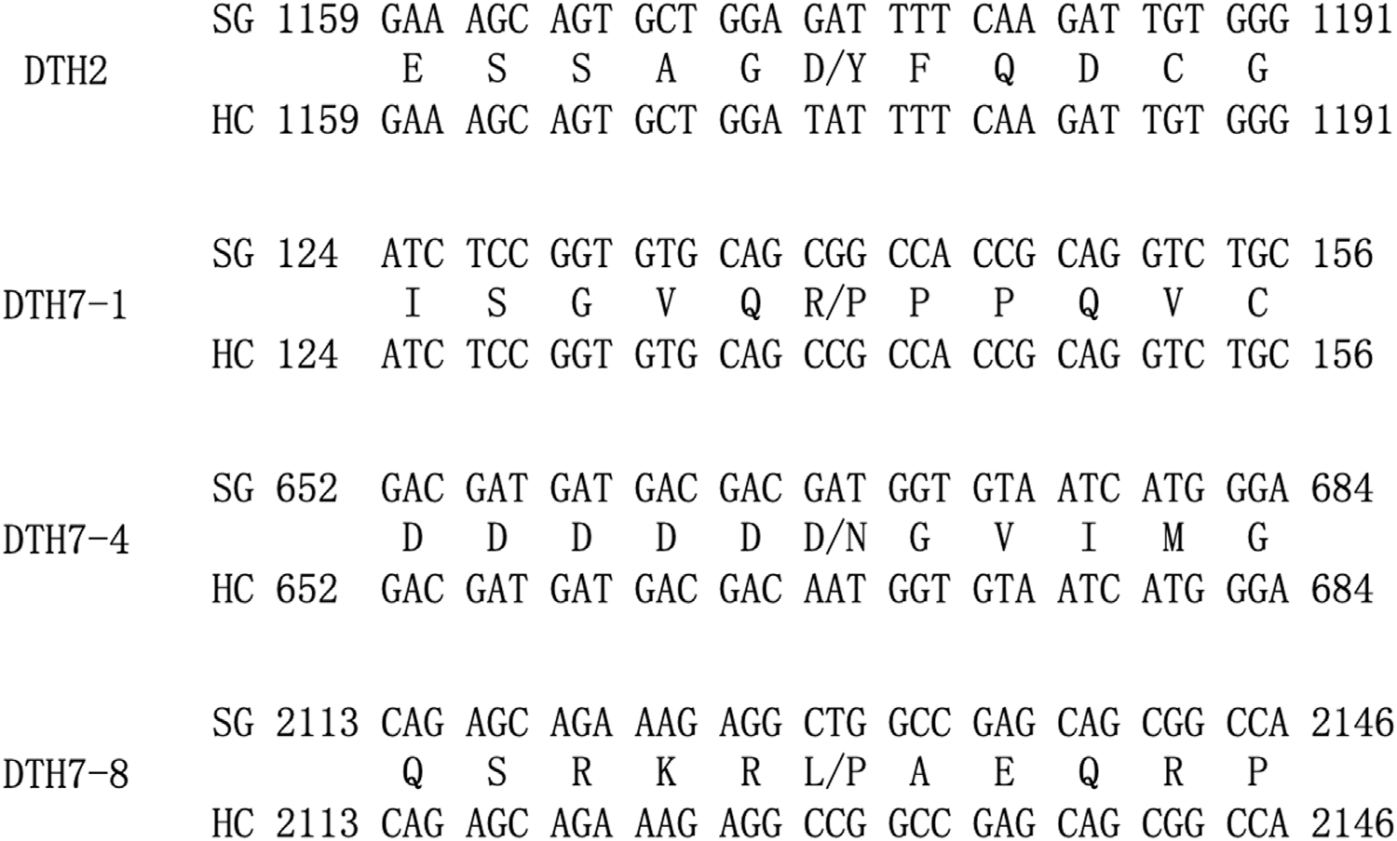
Schematic diagram of mutations in the bases of exons in the *DTH2* and *DHT7* genes and sequence variation of the coded proteins. On the left are gene names; in the upper row on the right are DNA sequences from SG; in the lower row on the right are DNA sequences from HC; and in the middle are coded proteins. Figures in front of and behind the sequence indicate the start and end of the relevant gene in the CDS region.

Genes were retrieved for the associated regions identified by the association analysis. Some genes in these regions have already been cloned (Table 2). These genes were sequenced and aligned to the reference genome to verify their polymorphism and differences in the coded proteins in parents. GAT/TAT mutation was found at base 1,174 of exon 3 in the *DTH2* gene on chromosome 2 in parents, resulting in the Asp/Tyr mutation of the coded protein. On chromosome 7, single-base mutations CGG/CCG, GAT/AAT, and CTG/CCG occurred in the exons 1, 4, and 8 of the *DTH7* gene, respectively. All three single-base mutations led to changes in the coded proteins, Arg/Pro, Asp/Asn, and Leu/Pro (Figure 3). The above results indicated that these two known genes at the genotype-associated loci on chromosomes 2 and 7 might be responsible for phenotypic changes. These associated regions on chromosomes 2 and 7 can be excluded from further analysis, while more efforts can be devoted to mining new genes in other associated regions.

### Mining and annotation analysis of candidate genes

**FIGURE 4:**
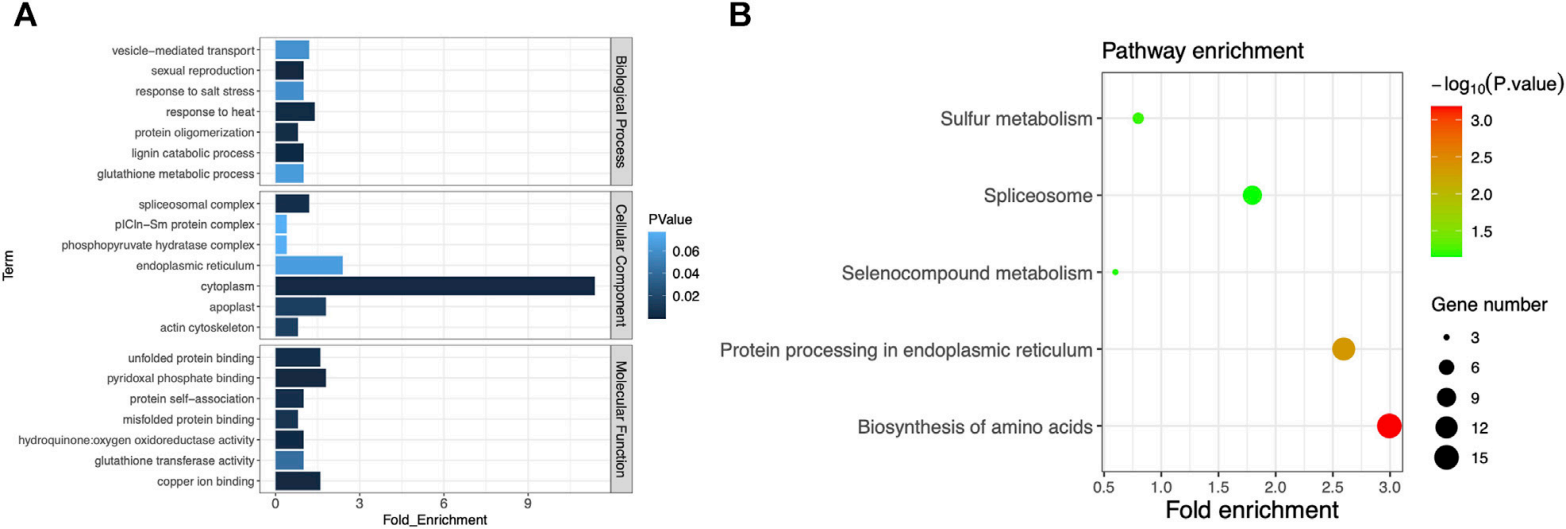
**(A)** GO cluster plot based on the functional enrichment analysis. The *y*-axis represents the GO term, and the *x*-axis represents the number of genes enriched in the GO term. Column color changes from dark to light, indicating the increase in the *p*-value of each item. **(B)** Functional analysis of KEGG pathways. The *y*-axis represents the name of the KEGG pathway, and the *x*-axis represents the fold enrichment of the gene. The size of the dot is based on the gene count enriched in the pathway, and the color of the dot shows the pathway enrichment significance (the darker the color, the higher the *p*-value).

With the associated regions on chromosomes 2 and 7 being excluded, the remaining associated regions contained 1,299 genes in total. Some of them may be the key genes associated with plant height or heading date, so we conducted the pathway analysis for further understanding of these genes. After ID conversion, 1,245 proteins were retrieved from the UniProt database. In the DAVID database, 502 DAVID IDs were retrieved in total. Among them, 255 proteins were annotated to GO category biological process, accounting for 50.8% of the total. These proteins belonged to 24 GO terms; 284 proteins were annotated to GO category cell component, accounting for 56.6%. These proteins belonged to seven GO terms; 281 proteins were annotated to GO category molecular function, accounting for 56%. These proteins belonged to 13 GO terms. The largest number of proteins was annotated to the GO term cytoplasm of the category cell component, totaling 57. The second-largest number of proteins were annotated to the GO term endoplasmic reticulum, totaling 12 (Figure 4A). The above results suggest that these protein-coding genes can play important roles in phenotypic regulation and deserve extra attention in further gene mining. By KEGG enrichment analysis, 119 genes were annotated to regulatory pathways, accounting for 23.7% of the total. There were five regulatory pathways for which more than two genes were annotated. The largest number of proteins (15) was annotated to the pathway “biosynthesis of amino acids.” Thirteen proteins were annotated to the pathway “protein processing in the endoplasmic reticulum” (Figure 4B; Supplementary Table S7). These proteins were very likely to have a direct impact on mutations in the days-to-heading or plant-height phenotype. Separate analysis is needed for each protein through a more refined experimental design.

## Discussion

Heilongjiang Province is considered the northernmost region among the rice cultivation zones of the world, with the degree of latitude ranging from 43°26′ to 53°33’ N. The region, thus located at high-latitude, varies little in effective accumulative temperature. The growth stage of rice cultivars grown in Heilongjiang Province ranges from 125 to 145 d (Wang et al., 2019). In addition, the photoperiod sensitivity of rice results in significant variation in the growth stage of rice cultivars grown in different regions. Precisely determining and utilizing the days to heading is of high importance to breed superior rice cultivars that fit with the so-called specific environment.

Cultivated rice is extensively grown in the world, which implies a great diversity of genes related to days of heading. Different genes and their alleles either positively or negatively regulate the regional adaptation of the cultivars. Therefore, understanding the functions and regulatory effects of each candidate gene in days to heading is of prime importance. So far, many genes related to days to heading have been cloned (Matsubara et al., 2014; Shrestha et al., 2014). The allelic variations of these genes in the cultivated rice population have drawn widespread attention. However, we still know little about the genetic diversity of rice cultivars in Heilongjiang, located in high-latitude regions. The precise gene regulatory effects in rice cultivars are largely unknown. The lack of knowledge makes it impossible to predict and assess the mature states of rice in a specific region based on an analysis of genes and their alleles related to days to heading. Precise molecular breeding of rice remains a great challenge. Constructing and studying the phenotypically segregated populations in different environments offers a pathway to mine unknown minor genes and their alleles. In this paper, we constructed the F_3_ segregating population consisting of 299 individuals, among which 10% were individuals with extreme phenotypes, totaling approximately 30. Two extreme phenotype pools were constructed using these individuals. The selected population was highly representative, which ensured the reliability of the QTL-seq results. The individuals in the population generally followed a normal distribution in terms of phenotypes. Transgressive segregation was observed among the individuals, indicating that the phenotypes of concern were regulated by multiple genes. The combination of different genes led to significant variations, which provides inspiration for further gene mining and breed improvement. However, due to the limitations in experimental conditions, the population size in our study was not large enough to investigate the phenotypic regulation by multiple genes. The differences between the two extremes of a phenotype were significant, resulting in the false positivity of some associated regions. Therefore, a large-scale near-isogenic line population should be constructed, and a precise phenotypic investigation is needed for mining minor genes in associated regions.

Most of the identified major genes related to days to heading in high-latitude regions have been readily applied to molecular breeding. These major genes can be used to regulate days to heading in different environments, but precise regulation is hardly impossible due to the large number of minor genes. After excluding the differences in major genes, a specific research methodology should be designed and applied to an appropriately selected study population to localize minor genes and facilitate their use in breeding. Furthermore, a variety of techniques have been developed and matured for gene localization in plants. For example, gene editing for mutant construction and single-cell transcriptome sequencing are combined with an appropriate study of populations to facilitate functional gene mining.

We identified 2,256 known genes in the shared regions associated with both plant height and days to heading phenotypes. However, gene ID conversion before annotation led to the missing of many gene IDs, and fewer genes were finally annotated than expected (Ashburner et al., 2000; Kanehisa et al., 2004). One reason might be the limited number of known proteins encoded by genes in these regions, which directly affects the prediction of associated regions. In addition, it is important that we establish reference genome data that are more precise, have larger coverage, and improve the existing genomic annotation databases so as to reduce gene loss during the annotation process. In this manner, we can expect to screen candidate genes more thoroughly in the associated regions using various techniques and experimental designs.

## Data Availability

The datasets presented in this study can be found in online repositories. The names of the repository/repositories and accession number(s) can be found in the article/Supplementary Material.
