# Supplementary material for: QTL-seq analysis identified the genomic regions of plant height and days to heading in high-latitude rice
Source: Front Genet. 2024 Feb 14;15:1305681. doi: 10.3389/fgene.2024.1305681 (PMC10899491; doi:10.3389/fgene.2024.1305681)
Supplement: Supplementary file 2 [file Table1.DOCX]

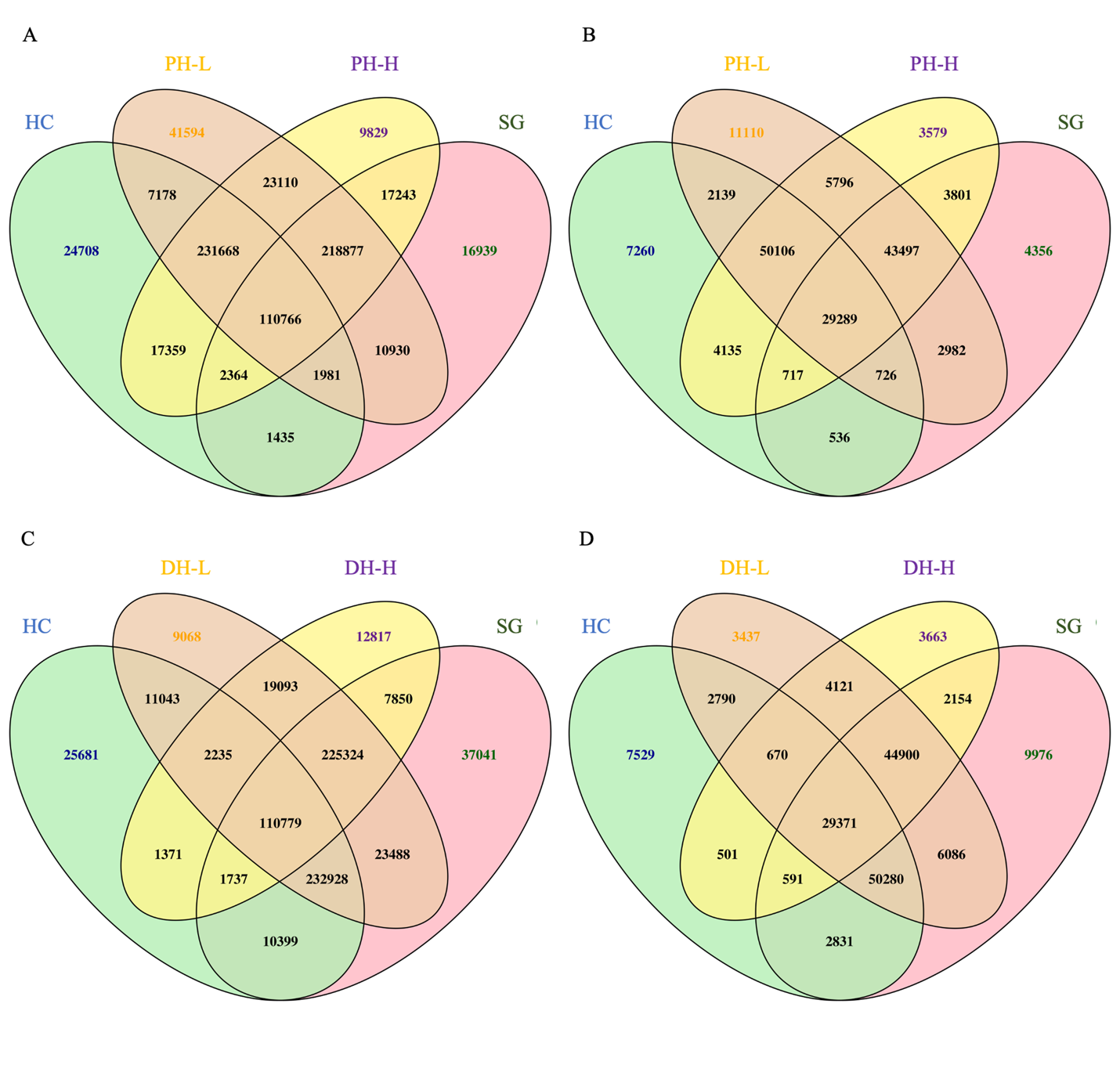


**Supplementary Figure S1** Venn diagram showing the statistics of mutation sites between the samples. (A) Statistics of SNPs in the four samples from the plant height genotype group. (B) Statistics of InDels in the four samples from the plant height genotype group. (C) Statistics of SNPs in the four samples from the days to heading genotype group. (D) Statistics of InDels in the four samples from the days to heading genotype group.


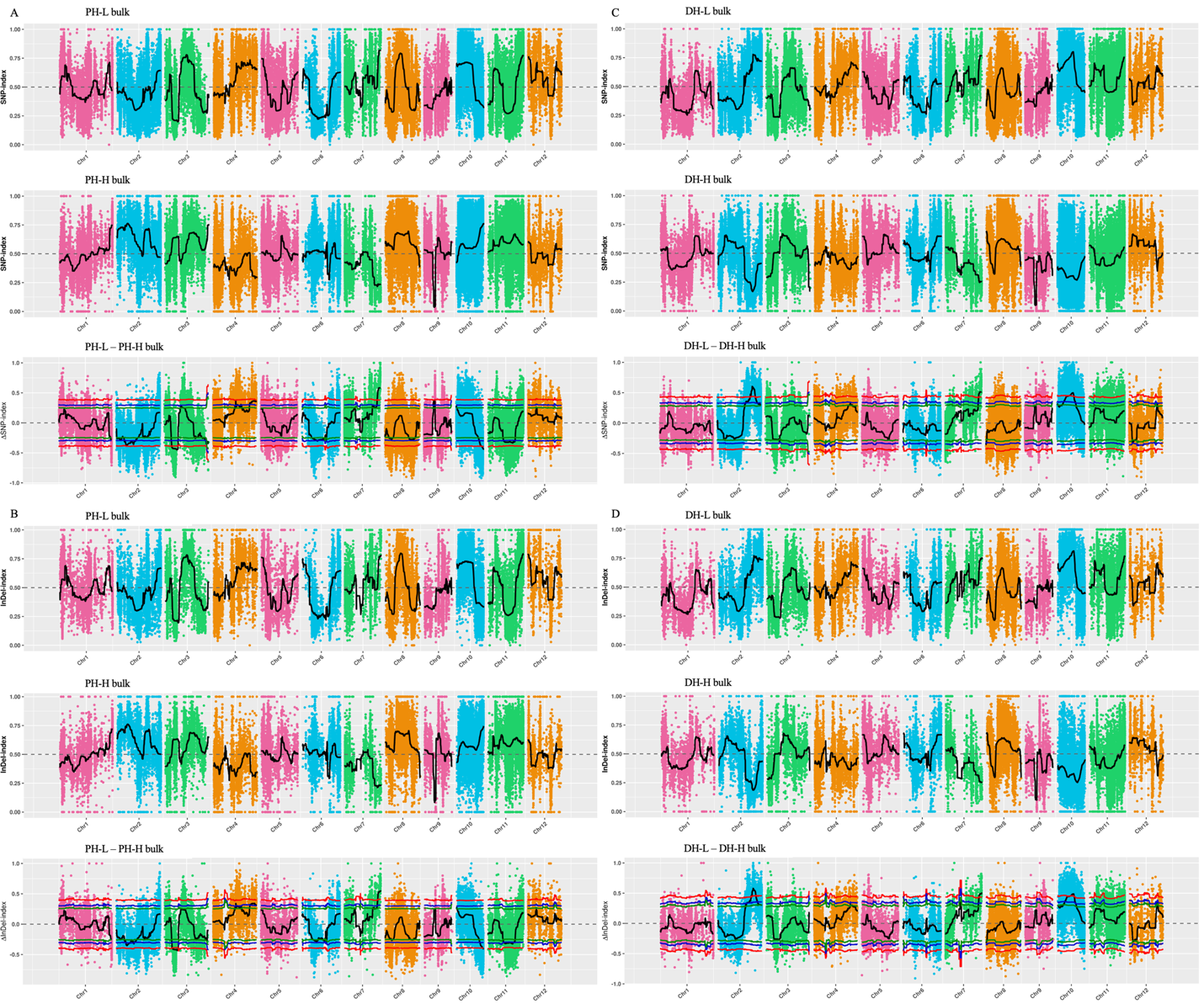


**Supplementary Figure S2** Distribution of SNP-index values at each locus on the chromosome. (A) SNP-index (or ΔSNP-index) values of each SNP associated with plant height genotype; (B) SNP-index (or ΔSNP-index) values of each small InDel associated with plant height genotype; (C) SNP-index (or ΔSNP-index) values of each SNP associated with days to heading phenotype; (D) SNP-index (or ΔSNP-index) values of each small InDel associated with days to heading phenotype. From top to bottom in each figure are the distributions of SNP-index values of the recessive phenotype pool and SNP-index values and ΔSNP-index values of the dominant phenotype pool successively. The x-axis represents the name of each chromosome; the colored dots represent the SNP-index (orΔSNP-index) values of each locus. The black line is the fitted SNP-index (or ΔSNP-index) value. The red line represents the threshold at a confidence level of 0.99; the blue line represents the threshold at the confidence level of 0.95; the green line represents the threshold at the confidence level of 0.90.
